# Overriding Surface Area Limitation: Mesopore-Driven Norfloxacin Adsorption on High-Temperature P-Doped Biochar

**DOI:** 10.3390/ma19173584

**Published:** 2026-08-24

**Authors:** Zhizhen Yin, Xizhen Yang, Nadilaimu Abudousuer, Xin Chen, Yuxin Liu, Jiayin Song

**Affiliations:** Key Laboratory of Pollutant Chemistry and Environmental Treatment, School of Resources and Environment, Yili Normal University, Yining 835000, China; yangxizhen2121@163.com (X.Y.); nadira0429@163.com (N.A.); chenxin112026@163.com (X.C.); liuyuxinn2026@163.com (Y.L.); sjy868788@163.com (J.S.)

**Keywords:** norfloxacin, P-doped biochar, adsorption

## Abstract

**Highlights:**

- 800 °C H_3_PO_4_ activation drastically reduces surface area (by ~90%) but creates mesopores (average 14.25 nm).
- Despite surface area loss, P-doped corn straw biochar removes >87% norfloxacin in 30 min (C_0_ = 50 mg·L^−1^; capacity up to 305.5 mg·g^−1^).
- Synergy of mesopore-dominated diffusion, graphitic π-π sites, and P-anchored chemisorption overrides specific surface area.

**Abstract:**

Antibiotic wastewater pollution is a serious environmental problem. This study prepared three types of P-doped biochar (P-CB, P-WB, and P-BB) from corn straw (CB), wheat straw (WB), and bamboo (BB) using phosphoric acid activation at 800 °C. Characterization by SEM, XRD, FTIR, and N_2_ adsorption confirmed that high-temperature H_3_PO_4_ activation drastically reduced the specific surface area but transformed the original microporous framework into a mesopore-dominated structure (average pore diameter 12–15 nm). Adsorption experiments showed that all P-doped biochars enhanced norfloxacin (NOR) removal, with P-CB performing best: 87.14% removal within 30 min at C_0_ = 50 mg·L^−1^ and a maximum adsorption capacity of 305.5 mg·g^−1^ at higher concentration. Kinetics followed a pseudo-second-order model (R^2^ > 0.9982), and isotherms fitted the Freundlich model (R^2^ = 0.9764–0.9855). Thermodynamics indicated a spontaneous and exothermic process, suggesting that the macroscopic driving force is dominated by physisorption. Mechanism analysis revealed that the synergistic effect of mesopore-dominated diffusion, graphitic π-π sites (from enhanced aromatization at 800 °C), and P-anchored chemisorption overrides the loss of specific surface area, enabling rapid and high-capacity adsorption. Optimal adsorption occurred at pH 5–9, while coexisting anions had a mild inhibitory effect. These findings demonstrate that high-temperature P-doping offers a strategy to rebalance pore architecture and surface functionality rather than simply maximizing specific surface area for efficient antibiotic removal from wastewater.

## 1. Introduction

The extensive use of antibiotics in both medical and livestock industries has led to their continuous input into the environment, posing a serious threat to ecosystems and human health [1,2]. As a typical representative of the third generation fluoroquinolone antibiotics, norfloxacin (NOR) is widely used due to its broad spectrum antibacterial activity [3,4]. Its residues in the environment can induce bacterial resistance genes [5,6], interfere with key processes such as nitrogen cycling in ecosystems [7], and exhibit chronic toxic effects on aquatic organisms [1]. Therefore, developing efficient, economical, and environmentally friendly technologies for antibiotic wastewater treatment has become a research hotspot in the environmental field [8].

Among various antibiotic removal technologies, the adsorption method is regarded as a highly promising treatment approach due to its simple operation, low cost, and the absence of toxic byproducts [9,10]. Biochar, as a carbon rich material produced by the pyrolysis of biomass under limited oxygen conditions, has attracted much attention in the field of wastewater treatment due to its large specific surface area, rich functional groups, wide raw material sources, and low preparation cost [11,12]. Studies have shown that biochar has a good adsorption capacity for antibiotic pollutants, and its adsorption mechanism involves various pathways such as π-π electron donor–acceptor interaction, hydrogen bonds, electrostatic interactions, and hydrophobic interactions [8,13,14,15].

However, the pore structure of the original biochar is mainly composed of micropores (smaller than 2 nm), with a single pore size distribution. This narrow pore channel restricts the diffusion and accessibility of macromolecular organic pollutants. At the same time, the number of surface active functional groups is limited, which restricts the efficient removal of polar pollutants [16,17].

To solve this problem, researchers have attempted to enhance the adsorption performance of biochar through various physical and chemical modification methods. Among them, phosphoric acid (H_3_PO_4_) activation is an efficient and commonly used chemical modification method [18,19,20,21]. Traditional phosphoric acid activation is usually carried out at a medium temperature range from 400 to 600 °C to promote the retention of phosphorus functional groups and the development of mesoporous structure. However, this study selects a high temperature pyrolysis condition of 800 °C, aiming to simultaneously achieve the improvement of the ordered degree of the carbon structure of biochar and the stable doping of phosphorus elements. The high temperature condition is conducive to the aromatization of the carbon skeleton and the enhancement of π electron cloud density, thereby strengthening π-π interactions. At high temperatures, phosphorus elements are stably anchored in the carbon matrix in the form of phosphate or phosphate ester, giving the material persistent surface chemical activity. Phosphoric acid activation can not only form developed mesoporous structures on the surface of biochar through etching, but also introduce phosphorus functional groups, significantly enhancing the electrostatic interaction, coordination interaction and π-π interaction between biochar and pollutants [22,23,24,25].

In recent years, phosphorus-modified biochar has demonstrated excellent performance in removing various pollutants from water bodies. For instance, Xie et al. (2023) used H_3_PO_4_ functionalized fish-scale biochar to adsorb diclofenac sodium and found that its extremely efficient adsorption ability was mainly attributed to the synergistic effect of phosphorus-containing functional groups [26]. Jaiswal et al. (2025) confirmed that the oxygen-containing functional groups on the surface of acid-modified biochar significantly enhanced the removal efficiency of arsenic [24]. Chen et al. (2018) studied the adsorption behavior of H_3_PO_4_-modified rice straw and pig manure biochar for tetracycline and found that the adsorption capacity of the modified biochar significantly increased, mainly due to pore filling, π-π interactions, and interactions [8]. Research on sulfamethoxazole also confirmed that P-doped biochar achieved efficient removal through multiple mechanisms such as electrostatic attraction, hydrogen bonds, and pore filling [26,27,28,29].

Several studies have reported on the adsorption of antibiotics by phosphorus-modified biochar. However, existing studies mostly focus on the evaluation of the adsorption performance of single raw materials or single pollutants. There is still a lack of systematic comparison of the adsorption performance differences of phosphorus-modified biochar from different agricultural and forestry waste sources for norfloxacin. The underlying mechanisms also need to be deeply explored. In addition, the thermodynamic behavior during the adsorption process is not yet fully addressed. The interactive influence of environmental factors remains unclear. The quantitative analysis of the synergistic effects of multiple mechanisms is also insufficient.

Different biomass raw materials have different contents of cellulose, hemicellulose, and lignin. As a result, they will form differentiated pore structures and surface chemical properties during pyrolysis and activation. These differences further affect their adsorption behavior for target pollutants [30]. It is worth noting that the understanding of the evolution of surface functional groups of biochar before and after phosphorus modification still needs to be deepened. In particular, the mechanism of the cooperative effect between phosphorus-containing functional groups and nitrogen-containing functional groups in the adsorption process is still unclear.

Despite the growing interest in phosphorus-modified biochar for antibiotic removal, several knowledge gaps remain. First, systematic comparisons of P-doped biochar derived from different biomass feedstocks for NOR adsorption are scarce. Second, the underlying reasons for the often-observed decrease in specific surface area after high-temperature phosphoric acid activation and its impact on adsorption performance have not been mechanistically explained. Third, the synergistic contributions of mesopore architecture, graphitic domains, and P-anchored functional groups to the overall adsorption process remain quantitatively unclear. To address these gaps, we propose the following hypothesis: high-temperature (800 °C) phosphorus doping rebalances the pore architecture and surface functionality, thereby overriding the loss of specific surface area and achieving rapid, high-capacity NOR adsorption. The specific objectives of this study are as follows. (i) To prepare and compare three types of P-doped biochar from corn straw, wheat straw, and bamboo via H_3_PO_4_ activation at 800 °C. (ii) To systematically characterize the physicochemical properties of these materials and identify the key structural and surface chemical factors governing NOR adsorption. (iii) To evaluate the adsorption kinetics, isotherms, thermodynamics, and environmental factors influencing NOR removal, and to quantitatively analyze the contributions of different adsorption mechanisms. (iv) To elucidate the synergistic adsorption mechanism combining mesopore-accelerated diffusion, π-π interactions, and P-anchored chemisorption. By systematically addressing these objectives, this study is expected to provide both a theoretical foundation and technical guidance for the value-added utilization of agricultural wastes and the efficient treatment of antibiotic-contaminated wastewater.

## 2. Materials and Methods

### 2.1. Chemicals and Materials

Norfloxacin (NOR, C_16_H_18_FN_3_O_3_, 98%) was purchased from Shanghai Macklin Biochemical Co., Ltd. (Shanghai, China). Phosphoric acid (H_3_PO_4_, 85%), sodium bicarbonate (NaHCO_3_, 99%), and sodium sulfate (Na_2_SO_4_, 99%) were supplied by Tianjin Beilian Fine Chemicals Development Co., Ltd. (Tianjin, China). Sodium hydroxide (NaOH, 96%) was obtained from Tianjin Zhiyuan Chemical Reagent Co., Ltd. (Tianjin, China). Hydrochloric acid (HCl, 37%) was purchased from Chengdu Colon Chemicals Co., Ltd. (Chengdu, China). Sodium carbonate (Na_2_CO_3_, 99%) was provided by Tianjin Damao Chemical Reagent Factory (Tianjin, China). Sodium chloride (NaCl, 99%) was obtained from Fuchen Chemical Reagent Co., Ltd. (Tianjin, China). All chemicals were of analytical grade and used without further purification. Deionized water was used in all experiments.

### 2.2. Preparation of Adsorbent

The feedstocks (corn straw, wheat straw, and bamboo) were washed with distilled water, dried at 110 °C for 2 h, crushed, and sieved through a 60-mesh screen. P-doped biochars were prepared via H_3_PO_4_ activation. A solution of H_3_PO_4_ and distilled water (2:1, *v*/*v*, 9 mL per 3 g biomass) was prepared under ultrasonication. The biomass powder (3 g) was added and stirred until homogeneous, then impregnated for 12 h. The mixture was pyrolyzed in a tube furnace at 800 °C for 2 h with a heating rate of 5 °C·min^−1^ under air atmosphere. After cooling, the product was ground, washed with deionized water via suction filtration until neutral pH (monitored by a pH meter), and dried at 60 °C. The resulting P-doped biochars were denoted as P-CB (P-doped corn straw biochar), P-WB (P-doped wheat straw biochar), and P-BB (P-doped bamboo biochar). For comparison, pristine biochars without H_3_PO_4_ activation were also synthesized and designated as CB (corn straw biochar), WB (wheat straw biochar), and BB (bamboo biochar), respectively.

The yields were determined gravimetrically as the mass ratio of the final biochar to the initial dry feedstock. The yields of CB, WB, BB, P-CB, P-WB, and P-BB were 35.6%, 33.1%, 30.2%, 34.2%, 31.8%, and 28.5%, respectively. The comparable yields indicate that H_3_PO_4_ treatment does not cause additional drastic mass loss at 800 °C, supporting the economic feasibility of this preparation route.

### 2.3. Characterization

The surface morphology and elemental distribution of the samples were observed using a scanning electron microscope (SEM, Sigma 300, ZEISS, Oberkochen, Germany). An X-ray diffractometer (XRD, Bruker, Karlsruhe, Germany) was used to analyze the crystal structure of biochar over a 2θ range of 10–80° with a step size of 0.4°, to investigate the crystalline phase and structural composition. Nitrogen adsorption–desorption isotherms were measured using an automatic surface area and porosity analyzer (Autosorb IQ, Quantachrome, Boynton Beach, FL, USA), and the specific surface area was calculated by the Brunauer–Emmett–Teller (BET) method. The surface functional groups of biochar were characterized by Fourier transform infrared spectroscopy (FTIR, Spectrum One, PerkinElmer, USA) in the wavenumber range of 4000–500 cm*^−^*^1^. Thermogravimetric analysis was performed using a simultaneous thermal analyzer (TGA/DSC1, Mettler Toledo, Greifensee, Switzerland) from 30~500 °C under N_2_ atmosphere to evaluate the thermal stability of the samples. X-ray photoelectron spectroscopy (XPS, K-Alpha, Thermo Scientific, Waltham, MA, USA) was used to investigate the surface elemental composition and chemical states of the materials.

### 2.4. Batch Adsorption Experiments and Adsorption Models

#### 2.4.1. General Batch Adsorption Procedures

The NOR stock solution (200 mg·L*^−^*^1^) was prepared by dissolving norfloxacin in deionized water and diluting to desired concentrations for subsequent experiments. For the adsorption experiments, an appropriate volume of NOR stock solution was diluted to 70 mg·L*^−^*^1^ with deionized water in a 50 mL conical flask. A certain amount of adsorbent was added to the solution. The mixture was stirred magnetically, and samples were taken at fixed time intervals to determine the residual NOR concentration using a UV-visible spectrophotometer (UV-5800PC, Shanghai Yuanxi Analytical Instruments Co., Ltd., Shanghai, China) at 280 nm. The adsorption capacity at time t (q_t_, mg·g^−1^), equilibrium adsorption capacity (q_e_, mg·g^−1^), and removal efficiency (R, %) were calculated by the following equations:

(1) $$q_{t}\;=\;\frac{{(C}_{0}\;-{\;C}_{t})\;\times \;V}{M}$$

(2) $$q_{e}=\frac{{(C}_{0}-{\;C}_{e})\;\times \;V}{M}$$

(3) $$R=\frac{{(C}_{0}-C_{e})}{C_{0}}\;\times \;100\%$$

where C_0_, C_t_ and C_e_ represent the initial concentration, the concentration at time t and the equilibrium concentration of NOR, respectively. M (g) is the mass of the adsorbent, and V (L) is the volume of the NOR solution.

#### 2.4.2. Adsorption Kinetic

Kinetic experiments were conducted by mixing 10 mg of adsorbent with 50 mL of NOR solution (70 to 170 mg·L^−1^). Samples were collected at 5, 10, 15, 20, 25, and 30 min. The experimental data were fitted using the pseudo-first-order (PFO), pseudo-second-order (PSO), Elovich, and intraparticle diffusion models. The key parameters for each model include the rate constants (k_1_, k_2_, K_id_), the initial adsorption rate constant (α), and the desorption constant (β). The full mathematical expressions of these models are provided in Section S1 (Equations (S1)–(S4)).

#### 2.4.3. Adsorption Isotherm Experiments

Isotherm experiments were conducted by mixing 10 mg of the optimal adsorbent with 50 mL of NOR solutions at initial concentrations ranging from 70 to 170 mg·L^−1^. The mixtures were shaken at 298, 308, and 318 K for 90 min to reach equilibrium. The equilibrium adsorption data were fitted using the Langmuir, Freundlich, Sips, and Temkin isotherm models, with the detailed mathematical expressions and parameter definitions provided in Section S1 (Equations (S5)–(S8)).

#### 2.4.4. Thermodynamic Experiments

Thermodynamic experiments were conducted by adding 10 mg of P-CB to 50 mL of NOR solution (70 mg·L^−1^) at 298, 308, and 318 K. The standard Gibbs free energy change (ΔG°), enthalpy change (ΔH°), and entropy change (ΔS°) were calculated using the van’t Hoff approach. Notably, the apparent distribution coefficient Kd = q_e_/C_e_ (L·g^−1^) is dimensional and cannot be directly used for thermodynamic calculations. Instead, the dimensionless thermodynamic equilibrium constant K° was derived from the Langmuir isotherm constant KL (L·mg^−1^). The corresponding thermodynamic equations and detailed derivation are provided in Section S1 (Equations (S9)–(S11)).

### 2.5. Regeneration and Reusability Experiments

The regeneration and reusability of P-CB were evaluated through consecutive adsorption/desorption cycles. In each cycle, 10 mg g of P-CB was added to 50 mL of NOR solution (70 mg·L^−1^) and the mixture was stirred magnetically for 1 h at room temperature. After adsorption, the adsorbent was separated by centrifugation, washed with deionized water, and dried at 60 °C. The recovered adsorbent was then immersed in 50 mL of HCl solution (0.1 mol·L^−1^) for 2 h under stirring to desorb the adsorbed NOR. Following desorption, the adsorbent was washed repeatedly with deionized water until the filtrate reached neutral pH, and then dried at 60 °C for the next adsorption cycle. This procedure was repeated for three consecutive cycles.

## 3. Results and Discussion

### 3.1. Characterization of Biochar

#### 3.1.1. Microscopic Morphology Analysis (SEM)

**Figure 1:**
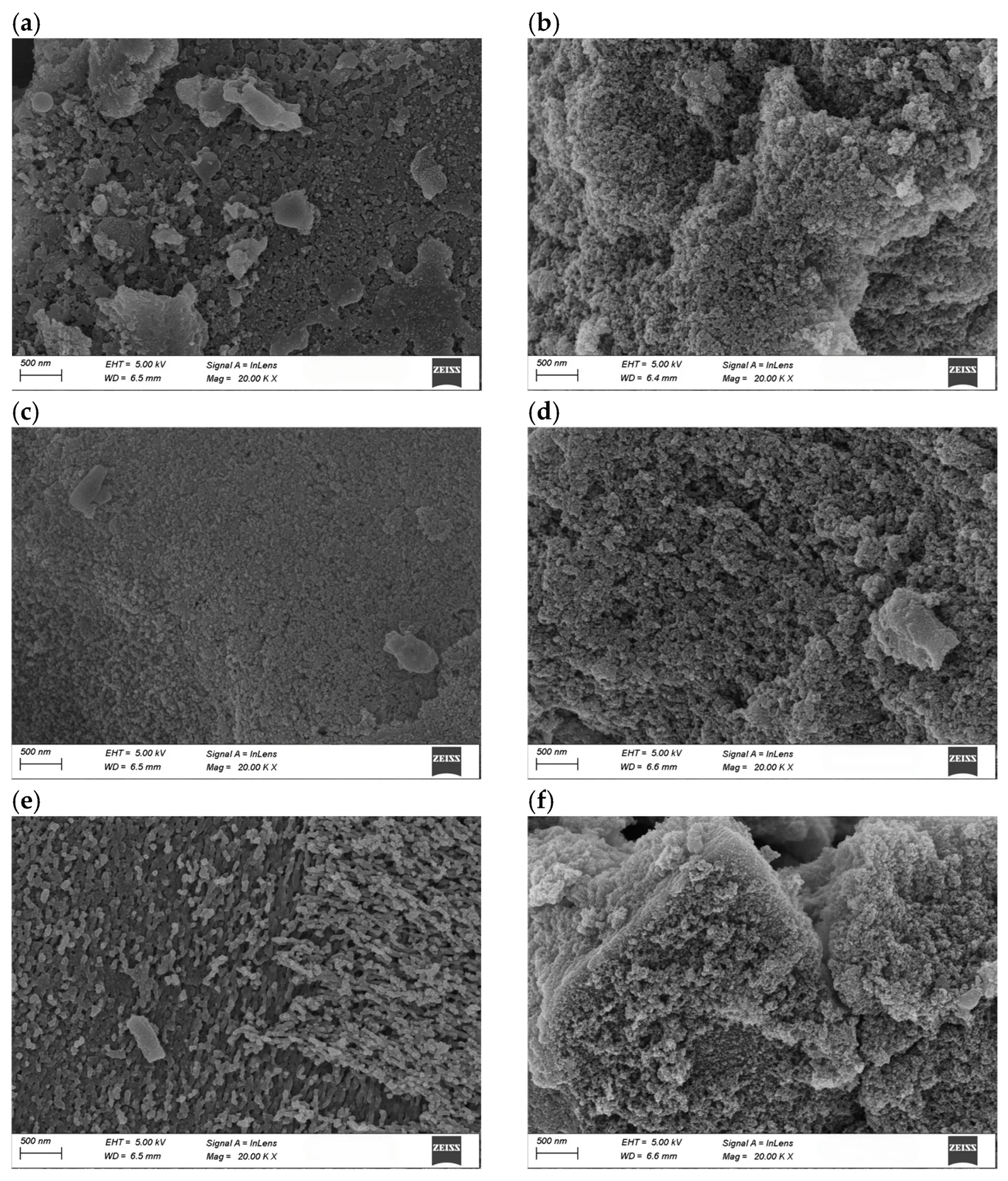
Scanning electron microscopy (SEM) images of BB (**a**), P-BB (**b**), WB (**c**), P-WB (**d**), CB (**e**), and P-CB (**f**).

Scanning electron microscopy (SEM) is used to observe the surface morphology and micrometer-scale pore structure of biochar. As shown in Figure 1, the surface of the original biochar is relatively smooth and compact, with limited observable macropores and mesopores on the surface. Only a few fibrous structures can be seen. This is the typical morphology of lignocellulosic biomass carbon after thermal decomposition [31].

After phosphoric acid activation, the surfaces of the three modified biochars (P-CB, P-WB, and P-BB) become extremely irregular and rough. They present a large number of broken structures and micrometer-scale grooves and depressions. This is due to the chemical etching and pore formation of phosphoric acid during the thermal decomposition process. This structural evolution helps to increase the specific surface area and expose more active sites. It indicates that phosphoric acid modification effectively enhances the pore structure of biochar [32].

It should be noted that SEM is limited by resolution. Therefore, it is difficult to directly observe micropores and some smaller mesopores (20–30 nm).

#### 3.1.2. XRD Analysis (XRD)

**Figure 2:**
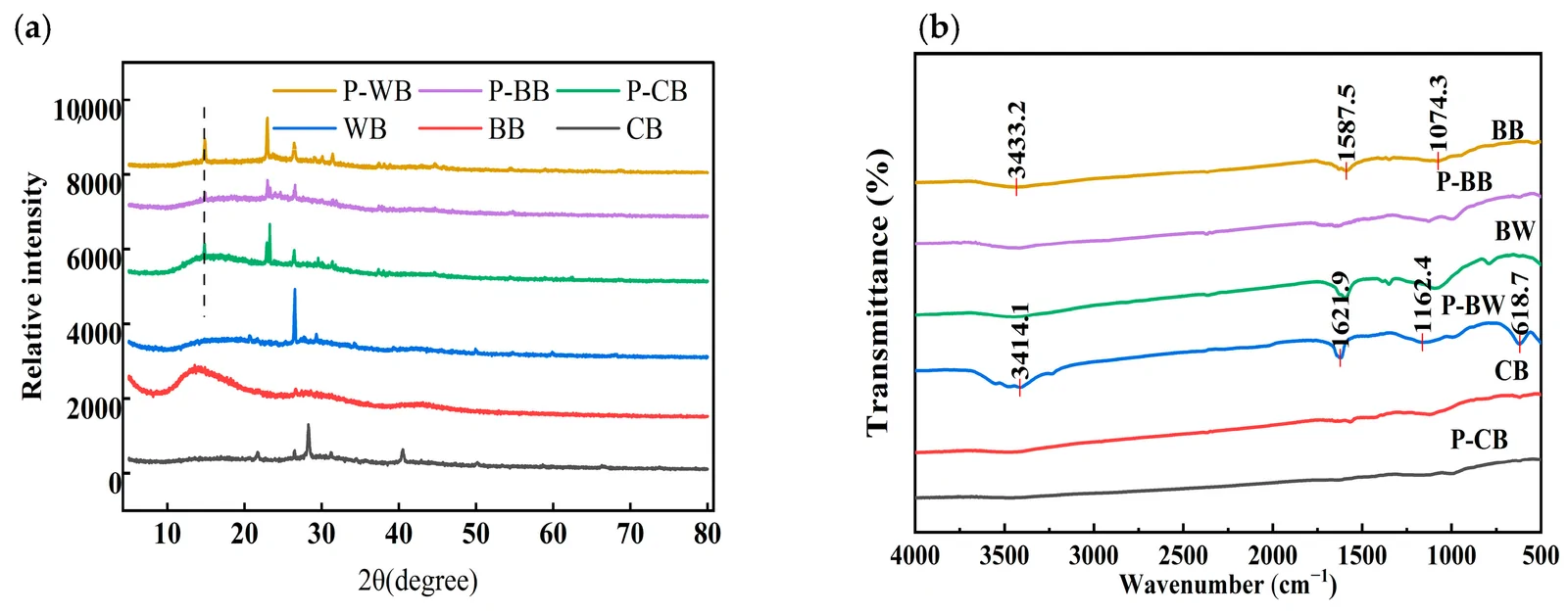
(**a**) XRD patterns and (**b**) FTIR spectra of pristine biochar and phosphoric acid-modified biochar.

The XRD patterns of the original biochar and the three phosphate-modified biochars are shown in Figure 2. The original biochar shows a broad peak at 2θ = 24°, indicating its amorphous carbon structure [33]. After phosphate modification, this peak is retained and its intensity is enhanced, suggesting that acidic oxygen functional groups have been successfully introduced into the crystalline region of the biochar. In addition, a well-crystallized phosphorus-containing phase has been formed [27,34].

The characteristic peak observed at 2θ = 26° corresponds to the reflection of the (002) crystal plane, which originates from parallel stacked graphene sheets. Notably, the peak intensity of the modified biochar on the (002) plane is slightly higher than that of the original biochar, indicating a higher degree of order in the carbon structure [35]. This higher order ensures a high π electron cloud density, which is conducive to π-π interactions in the subsequent adsorption process. At 2θ = 15°, the three phosphate-modified biochars all show minor shoulder peaks, which can be attributed to the carbonization of carbon within the polymer chains. Additionally, the addition of acidic oxygen groups leads to the breakage of the cellulose polymer chains, consistent with previous studies [26,27].

#### 3.1.3. Surface Functional Group Analysis (FTIR)

FTIR was used to characterize the surface functional group structures of the modified and unmodified biochars. Overall, the infrared absorption peak intensities of all biochar samples are relatively weak, mainly due to the high carbonization and aromatization of biomass during pyrolysis, which thermally removes most polar functional groups and orders the carbon framework while typically forming surface functional groups closely related to chemical adsorption [36].

**Figure 3:**
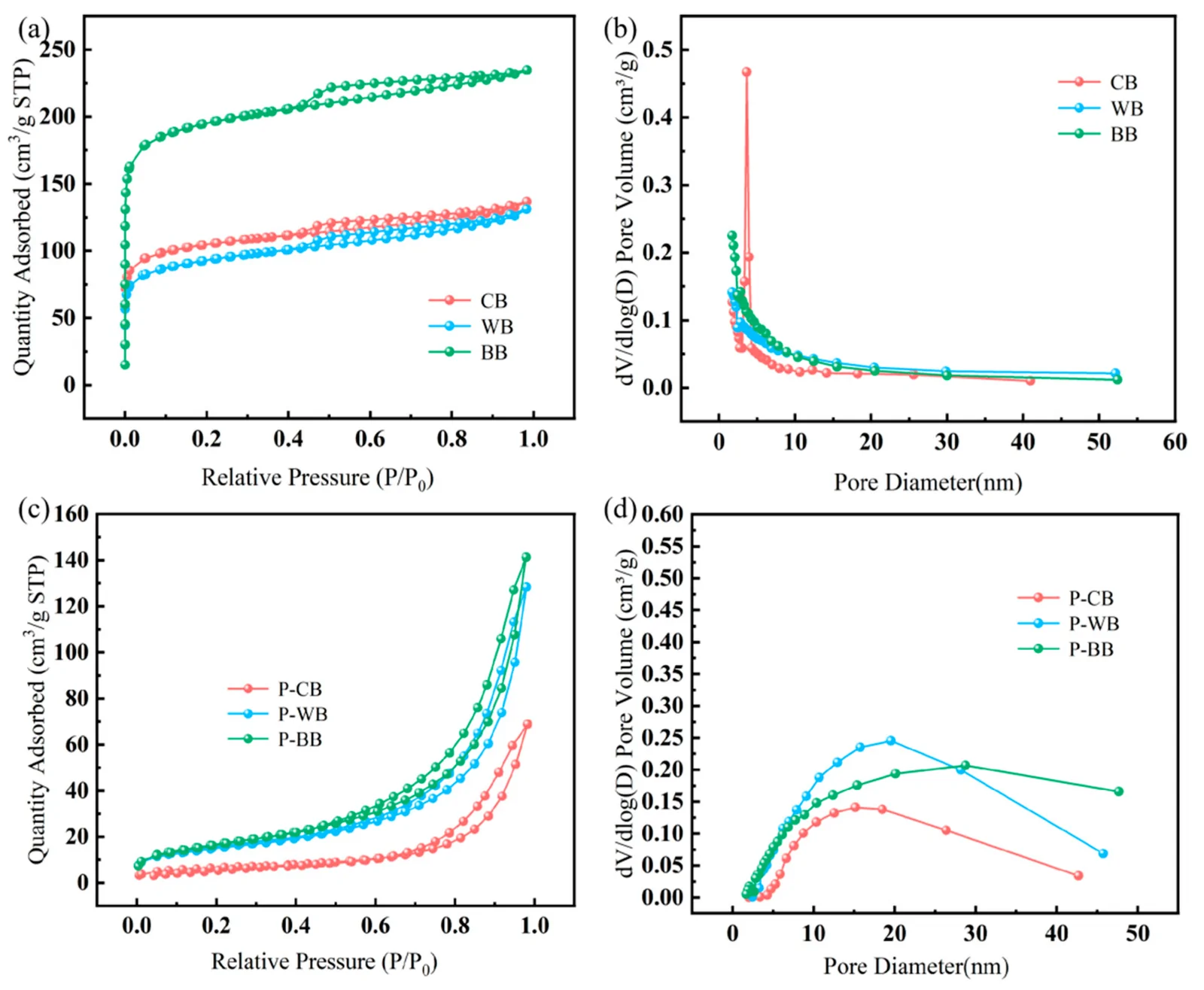
Pore structure characteristics of biochars: (**a**) N_2_ isotherms of pristine biochars, (**b**) BJH curves of pristine biochars, (**c**) N_2_ isotherms of P-doped biochars, and (**d**) BJH curves of P-doped biochars.

Figure 3 shows a broad and weak absorption peak at approximately 3414–3433 cm^−1^, belonging to the stretching vibration of adsorbed water and residual hydroxyl groups (–OH) [27,29,34]. The absorption peak near 1587 cm^−1^ corresponds to the C=C stretching vibration in the aromatic ring, a typical feature of carbon materials that form stable aromatic structures during pyrolysis [29]. Additionally, a relatively weak peak near 1074 cm^−1^ is attributed to C–O–C ether bonds or C–OH alcohol hydroxyl groups [37,38], and its low intensity suggests limited residual oxygen-containing functional groups. After phosphorus modification, the FTIR spectra of the three biochars are essentially unchanged. No typical P–O characteristic peaks are observed, mainly due to the strong infrared absorption of the highly aromatized carbon skeleton masking surface functional group signals. Therefore, FTIR has limitations for such highly graphitized carbon materials. In contrast, surface-sensitive techniques such as XPS can more effectively characterize the surface chemical states.

#### 3.1.4. BET Specific Surface Area and Pore Structure Analysis

The N_2_ adsorption–desorption isotherms and pore parameters of the six biochar samples are shown in Figure 3 and Table 1, respectively. According to the IUPAC 2015 classification, the pristine biochars (CB, WB, BB) exhibit Type IV(a) isotherms with H_4_ hysteresis loops, indicating the coexistence of micropores and slit-shaped mesopores. Their BET specific surface areas follow BB (748.18 m^2^·g*^−^*^1^) > CB (393.08 m^2^·g*^−^*^1^) > WB (345.43 m^2^·g*^−^*^1^), with micropore areas accounting for 89.2%, 88.7%, and 82.5% of the total BET specific surface areas, respectively.

For the P-doped biochars (P-CB, P-WB, P-BB), the N_2_ uptake decreases significantly compared with the pristine biochars, consistent with the reduced BET specific surface areas (23.56–61.31 m^2^·g*^−^*^1^). The isotherms exhibit Type IV(a) behavior with H_3_ hysteresis loops, indicative of mesoporous structures featuring slit-shaped pores. The BET specific surface area and micropore volume decrease significantly unpon P-doping; for instance, the BET specific surface area of BB decreases from 748.18 to 61.31 m^2^·g*^−^*^1^, and the micropore area proportion drops from 89.2% to 15.7% (Table 1). Three factors contribute to this pore structure transformation: (i) chemical etching by H_3_PO_4_ at 800 °C, which removes carbon atoms and widens micropores into mesopores; (ii) pore wall collapse during the subsequent washing step, particularly pronounced in bamboo-derived biochar (BB) due to its thin pore walls, explaining why P-BB shows the greatest surface area reduction (91.8%); and (iii) structural reconstruction, as the high-temperature treatment promotes the reorganization of the carbon skeleton into more thermally stable structures, which reduces the number of defective micropores while preserving mesopores. The BJH desorption average pore diameter increases from 3.31 nm (CB) to 14.25 nm (P-CB) and from 3.74 nm (WB) to 12.97 nm (P-WB), confirming the micropore-to-mesopore transformation [39]. Despite the significant decrease in BET specific surface area, the increased mesopore proportion is beneficial for reducing the diffusion resistance of large pollutant molecules [28,37,40].

#### 3.1.5. Surface Chemical Property Analysis (XPS)

**Figure 4:**
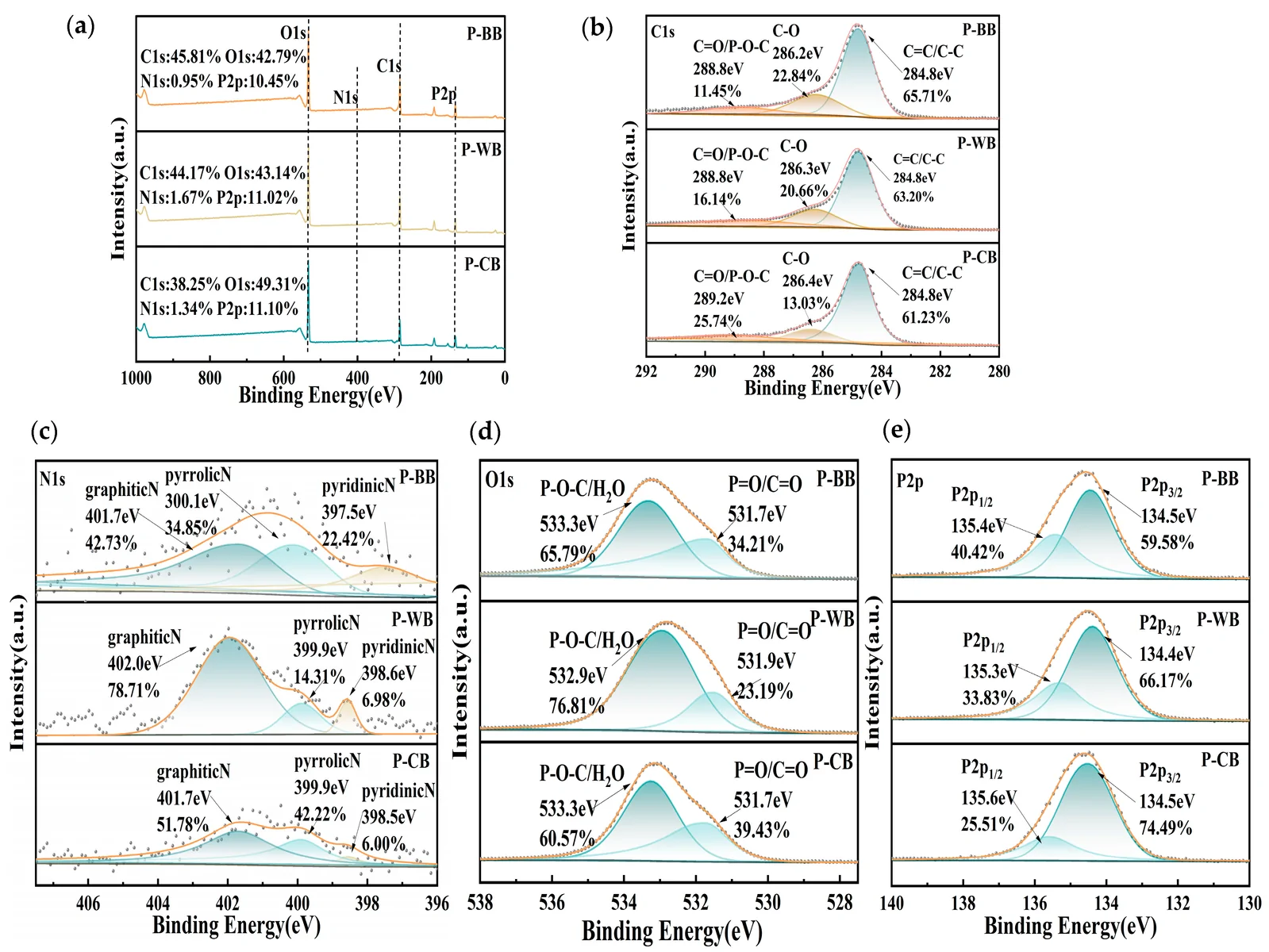
XPS spectra of P-CB, P-WB, and P-BB: (**a**) survey spectrum, (**b**) C 1s, (**c**) N 1s, (**d**) O 1s, and (**e**) P 2p.

The surface elemental composition and chemical state of the P-CB, P-WB, and P-BB samples were characterized by XPS. The full spectrum scanning results are shown in Figure 4a. All three materials contained four elements: C, N, O, and P. Among them, the O element content on the surface of the P-CB sample was 49.31%. The P element content was 11.10%. Both values were higher than those of P-WB and P-BB. Different raw materials were treated under the same phosphoric acid activation conditions. The differences in elemental content reflect the influence of the raw material’s chemical composition. This composition affects the phosphorus doping efficiency and the retention degree of functional groups. The high resolution spectra of C1s are shown in Figure 4b. All three materials exhibit three characteristic peaks near 284.8 eV, 286.4 eV, and 288.8 eV. These peaks correspond to C–C, C=C, C–O, and C=O or P–O–C bonds. This is consistent with the literature [29,41,42]. Compared to P-WB and P-BB, the peak proportion of C=O or P–O–C bonds in P-CB is relatively higher (25.74%). The binding energy is also higher (289.2 eV). The proportion of C–O bonds is relatively lower (13.03%). The N1s spectrum is shown in Figure 4c. It can be divided into three characteristic peaks. These peaks belong to graphite nitrogen (401.7 eV), pyrrole nitrogen (399.9 eV), and pyridine nitrogen (398.5 eV).

The proportion of graphite nitrogen in P-CB is 51.78%. The proportion of pyrrole nitrogen is 42.22%. Both proportions are relatively higher than those in P-BB. The O1s spectrum is shown in Figure 4d. It can be divided into two characteristic peaks. The peak at 533.3 eV belongs to the P–O–C bond or adsorbed water. The peak at 531.7 eV belongs to P=O or C=O bonds [43]. The P2p spectrum is shown in Figure 4e. It presents two characteristic peaks. These correspond to the P2p_3/2_ and P2p_1/2_ orbitals [40]. The binding energy range of P2p_1/2_ in P-CB is 135.3 to 135.6 eV. The range of P2p_3/2_ is 134.4 to 135.5 eV. These are attributed to –PO_3_ and –PO_4_, respectively [13]. The P2p_3/2_ binding energy of the –PO_4_ structure in P-CB is 134.5 eV, and this structure accounts for up to 74.49%. This confirms that the phosphorus element is mainly anchored on the carbon skeleton in a stable phosphate ester or phosphate salt form.

In conclusion, the P-CB surface is rich in C=O and P–O–C bonds (25.74%), pyrrole nitrogen (42.22%), and –PO_4_ structures (74.49%). It also has a high O content (49.31%). These surface chemical characteristics are expected to enhance the affinity for polar organic pollutants. This enhancement occurs through hydrogen bonds, complexation, electrostatic attraction, and π-π interactions [44].

### 3.2. Adsorption Performance Study

#### 3.2.1. Performance Analysis of Different Modified Biochars

**Figure 5:**
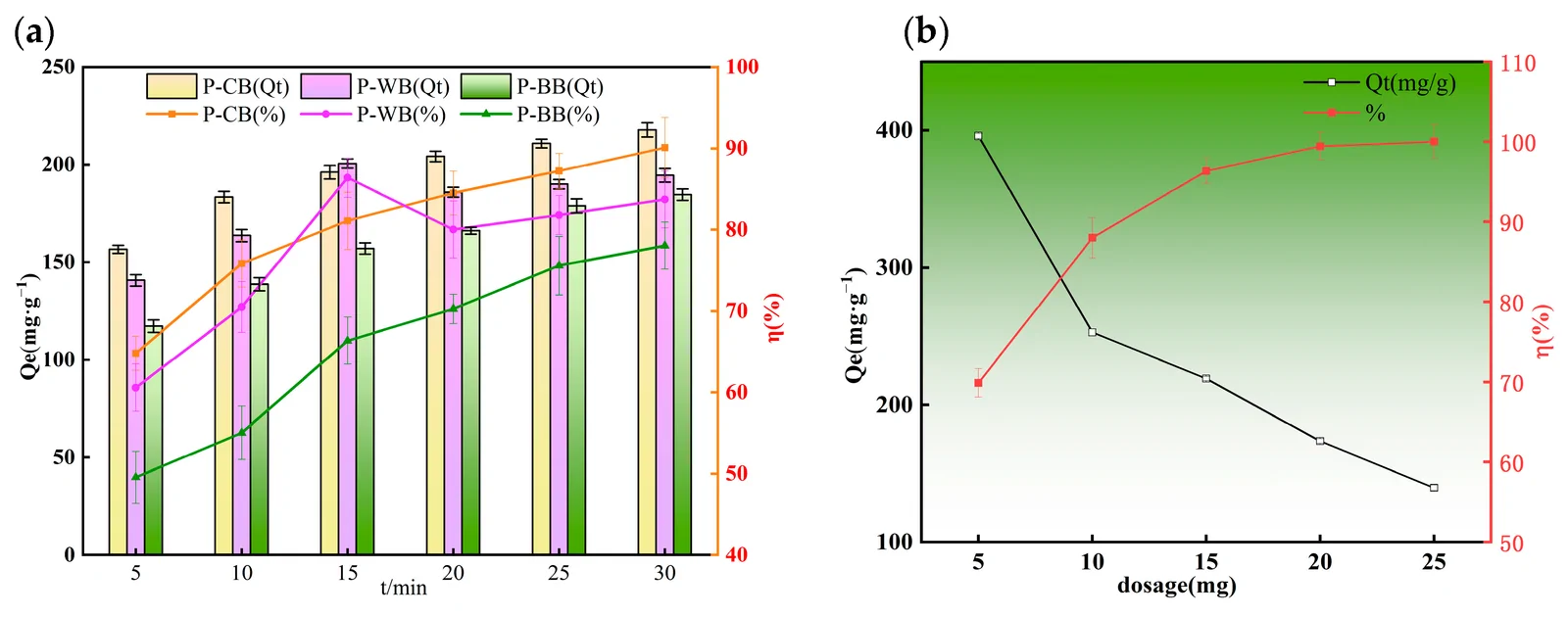
Adsorbent screening and dosage optimization: (**a**) comparison of NOR adsorption performance of different biochars (adsorbent dosage = 10 mg, C_0_ = 50 mg·L^−1^); (**b**) optimal dosage (C_0_ = 70 mg·L^−1^).

The adsorption performance of the original biochar and the phosphoric acid-modified biochar was compared. The experimental conditions were the same. The adsorbent dosage was 10 mg. The results are shown in Figure 5a.

This combined figure consists of two parts. The main figure shows the adsorption kinetics of the three phosphoric acid-modified biochars for NOR. The inset small figure in 5a presents a comparison. It compares the equilibrium adsorption amounts and removal rates of six materials at 30 min. The adsorption amounts of the three original biochars, CB, WB, and BB, were 30, 24, and 16 mg·g^−1^, respectively. The trends of adsorption amount and removal rate of the three modified materials with time were consistent. There was a rapid increase in the initial stage. Then, the increase rate became slower. Equilibrium was essentially reached within 30 min. The performance ranking was P-CB > P-WB > P-BB. After phosphoric acid modification, the adsorption amounts of P-CB, P-WB, and P-BB increased. They increased to 217.84, 194.46, and 184.63 mg·g^−1^, respectively. The removal rates reached 87.14%, 83.74%, and 77%. In contrast, the adsorption performance of the original biochars was relatively low. Among them, P-CB derived from corn straw showed the most outstanding performance. In conclusion, phosphoric acid modification significantly improved the adsorption performance of biochars from different raw materials. The effect was most prominent for corn straw biochar after modification.

Based on the characterization results, the excellent adsorption ability of P-CB was attributed to several factors. These included its mesoporous structure with an average pore diameter of 14.25 nm, its high surface phosphorus content (11.10%), and the synergistic effect of C=O, P–O–C bonds (25.74%) and pyrrole nitrogen (42.22%). Therefore, subsequent research selected P-CB as a representative adsorbent. It was used to conduct systematic investigations on adsorption kinetics, isotherms, thermodynamics, and environmental impact factors.

#### 3.2.2. Optimization of Adsorption Dosage

To prevent the rapid adsorption at low concentrations that would lead to insignificant differences in adsorption results, an initial NOR concentration of 70 mg·L^−1^ was selected to investigate the influence of different dosage levels on NOR adsorption performance. The results are shown in Figure 5b.

As the dosage increased from 5 mg to 25 mg, the NOR removal rate continuously rose from 69.9% to 100%. The unit adsorption capacity decreased from 395.86 mg·g^−1^ to 139.63 mg·g^−1^. Increasing the dosage provided more adsorption sites, thereby enhancing the removal efficiency. The phenomenon of decreasing adsorbent utilization rate with increasing dosage has also been reported in related studies [40,45].

Both the removal rate and the adsorbent utilization rate were considered. When the dosage increased from 5 mg to 10 mg, the removal rate increased from 69.9% to 77.0%, which was a significant increase. The adsorption capacity decreased to 251.35 mg·g^−1^, but it was still at a relatively high level. Further increasing the dosage led to a slower increase in the removal rate. The adsorption capacity continuously decreased, resulting in a significant reduction in adsorbent efficiency. Therefore, 10 mg was determined as the optimal dosage. At this dosage, the removal rate was 77.0% and the adsorption capacity was 251.35 mg·g^−1^. This dosage is convenient for optimizing the subsequent experimental conditions.

#### 3.2.3. Adsorption Kinetic Analysis

**Figure 6:**
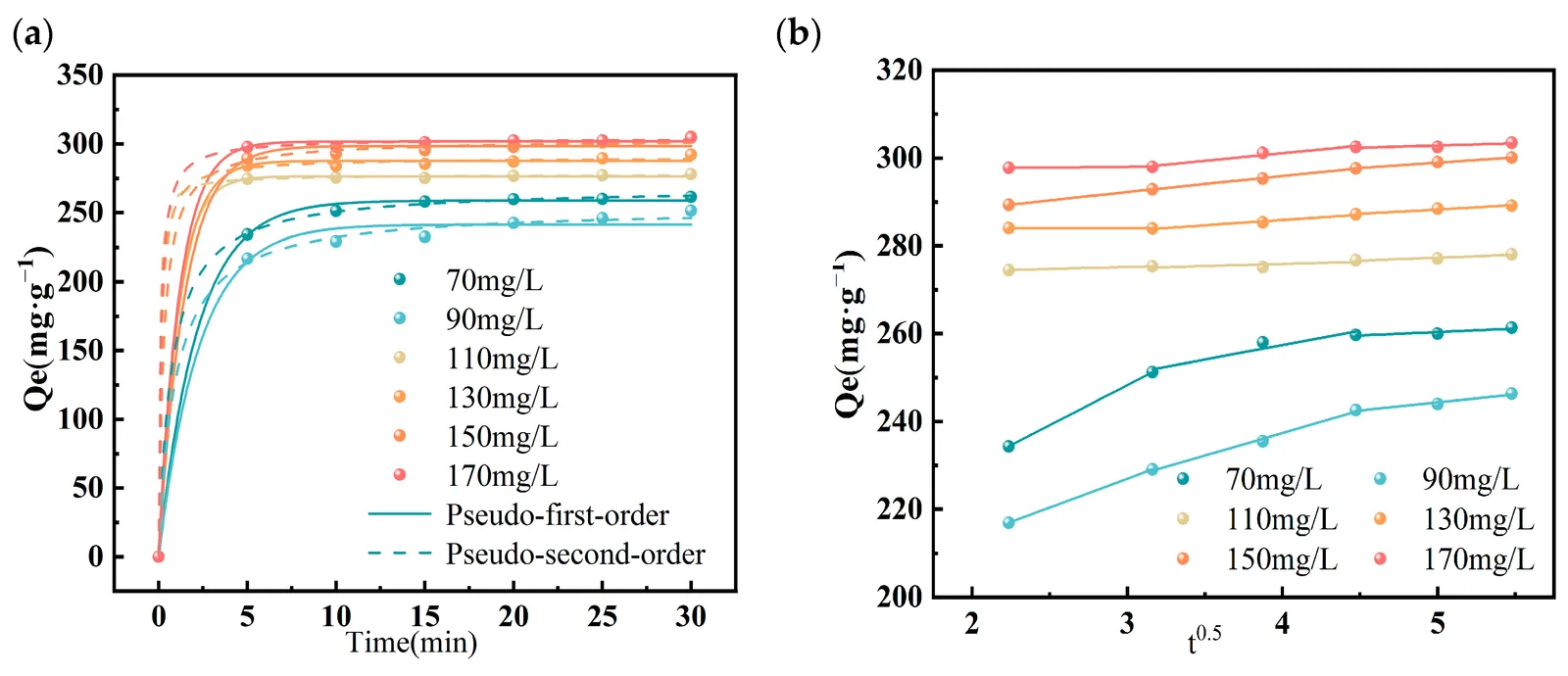
Adsorption kinetics fitting of NOR onto P-CB: (**a**) pseudo-first-order and pseudo-second-order nonlinear models, (**b**) intraparticle diffusion model.

To investigate the adsorption kinetic characteristics of P-CB for NOR, the changes in adsorption amount over time were examined at different initial concentrations (70–170 mg·L^−1^). The data were fitted using the pseudo-first-order (PFO), pseudo-second-order (PSO), and intraparticle diffusion models. The fitting parameters are presented in Table 2 and Table 3. Figure 6a shows that the PSO model (R^2^ > 0.9982) outperforms the PFO model (R^2^ = 0.87–0.95). Beyond the R^2^ comparison, the root mean square error (RMSE) was introduced as a quantitative indicator of model performance (Table S1). The RMSE values consistently show that the PSO model yields lower errors (0.796–4.214) than the PFO model (1.093–7.580) across all concentrations. For instance, at 70 mg·L^−1^, the RMSE of PSO (1.079) is less than half of that of PFO (2.671). The calculated equilibrium adsorption amount (*q_e,ca_*_l_) from the PSO model also shows a relative error of less than 3% compared to the experimental value (*q*_e,exp_). This reduction, together with the agreement between *q*_e,cal_(PSO) and *q*_e,exp_, confirms that chemisorption is the rate-controlling step [46,47,48]. The overall process is therefore controlled by the synergistic combination of physical transport and subsequent chemical binding. The complete statistical indicators, including adj. R^2^, reduced χ^2^, SSE, and RMSE, are provided in Table S1.

Figure 6b shows that the intraparticle diffusion model fitting presents a three-stage linear relationship. At most concentrations, the parameters show K_ip1_> K_ip2_ > K_ip3_ and C_1_ < C_2_ < C_3_, resolving the adsorption process into membrane diffusion, pore internal diffusion, and adsorption equilibrium. The fitting lines do not pass through the origin (C > 0), indicating that membrane diffusion is one of the rate-limiting steps, consistent with literature [18,49]. Other rate-limiting mechanisms include electrostatic interaction and ion exchange [50].

The two-stage adsorption behavior, with rapid uptake within the first 5 min followed by a slower stage, can be mechanistically interpreted as follows. Stage I (0–5 min) is governed by external mass transfer and mesopore-facilitated diffusion, where the mesopore-dominated architecture (average pore diameter 14.25 nm) provides efficient pathways with minimal steric hindrance. This stage accounts for over 80% of the equilibrium capacity. Stage II (5–30 min) is controlled by intraparticle diffusion into less accessible regions, combined with the transition from physical transport to chemical interactions (hydrogen bonding, π-π interactions, and P-anchored coordination), which require specific molecular orientation and proceed more slowly. This interpretation is consistent with the three-stage intraparticle diffusion model (Table 3), where the transition from K_ip1_ to K_ip2_ reflects the shift from mesopore/film diffusion to intraparticle diffusion control.

The initial adsorption rate (h) was calculated from the pseudo-second-order parameters (Table 2). The h values increase progressively with increasing initial NOR concentration, from 242.3 mg·g^−1^·min^−1^ at 70 mg·L^−1^ to 306.7 mg·g^−1^·min^−1^ at 170 mg·L^−1^, confirming that higher concentration enhances the mass transfer driving force [51].

### 3.3. Adsorption Mechanism Exploration

#### 3.3.1. Isothermal Adsorption Analysis

**Figure 7:**
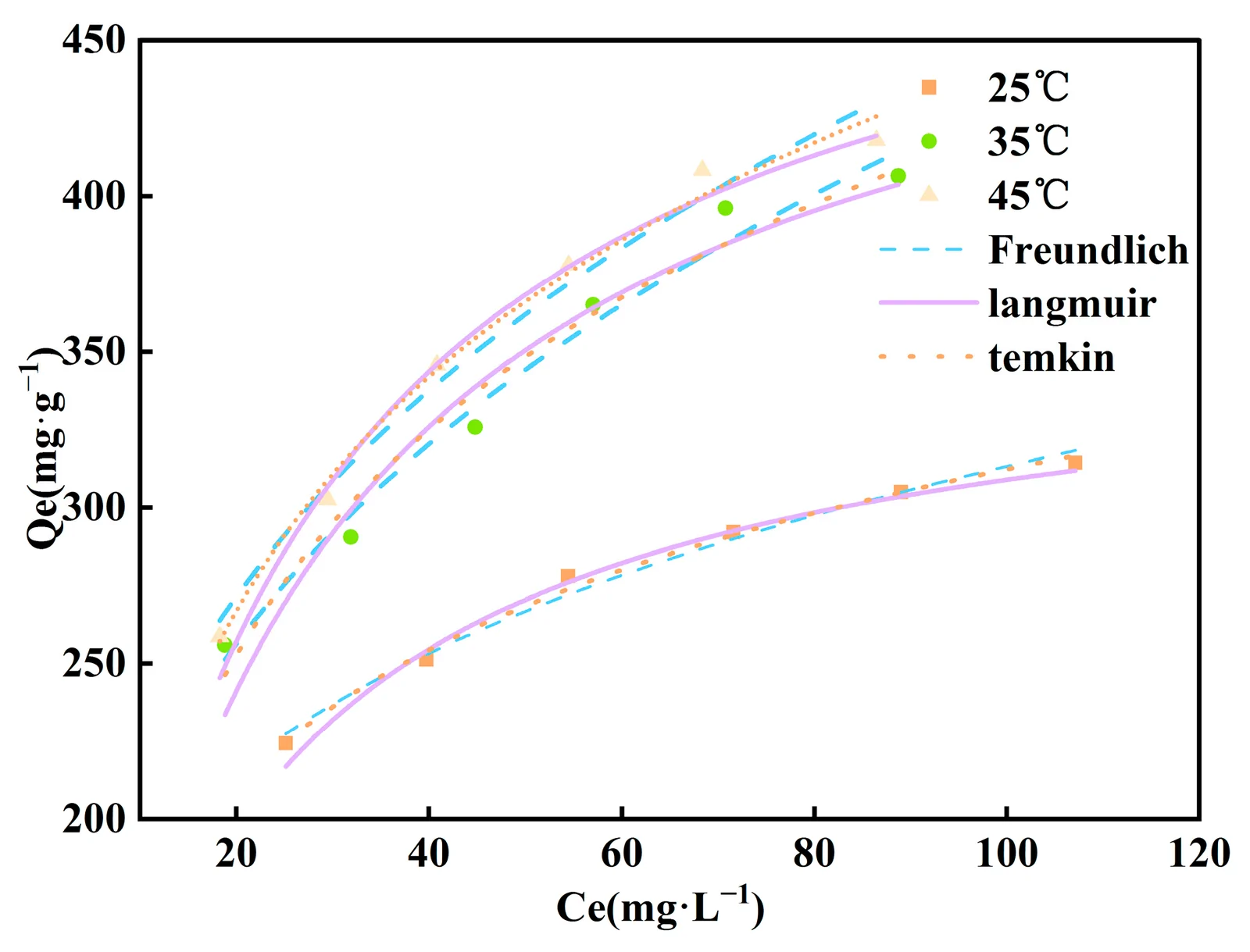
Adsorption isotherm model (adsorbent dosage = 10 mg, C_0_ = 70 mg·L^−1^).

To investigate the adsorption capacity and mechanism of P-CB for NOR, three isothermal adsorption models (Langmuir, Freundlich, and Temkin) were used to fit the equilibrium data at 298, 308, and 318 K. The results are shown in Figure 7 and the fitting parameters in Table 4. To quantitatively discriminate among the three models, the root mean square error (RMSE) was adopted as the primary goodness-of-fit indicator alongside R^2^.

The Freundlich model fits well at all temperatures (R^2^ = 0.9764–0.9855), with 1/n values ranging from 0.232 to 0.322 (all < 0.5), indicating strong adsorption affinity and significant surface heterogeneity. The Temkin model also yields good fits (R^2^ = 0.9768–0.9932), further confirming adsorbate–adsorbent interactions. This surface heterogeneity arises from abundant oxygen/phosphorus functional groups and graphite-like domains on P-CB, which provide heterogeneous active sites for multiple adsorption mechanisms [51,52,53,54,55].

The Langmuir model shows good fits (R^2^ = 0.9422–0.9766), suggesting monolayer coverage plays an important role. The maximum theoretical adsorption capacity (q_m_) increases with temperature from 352.26 mg·g^−1^ at 298 K to 511.94 mg·g^−1^ at 318 K. However, the Langmuir constant K_L_ does not show a monotonic trend, which may be attributable to experimental uncertainty associated with the limited number of temperature points employed in this study. The RMSE values in Table 4 reveal a temperature-dependent shift in model preference. At 298 K, the Temkin model exhibits the lowest RMSE (2.773), substantially lower than Langmuir (3.562) and Freundlich (4.116), indicating a relatively uniform adsorption energy distribution at room temperature. At 308 and 318 K, the Langmuir model yields the lowest RMSE (5.847 and 6.053, respectively), suggesting elevated temperatures promote homogeneous monolayer chemisorption. This temperature-dependent evolution, combined with the PSO kinetic model, suggests that chemisorption contributes to the adsorption mechanism, although the thermodynamic analysis indicates that the overall enthalpy change is exothermic. The Langmuir model is employed solely for estimating qm; mechanistic conclusions are based on the PSO model, thermodynamics, and XPS analysis.

The dimensionless separation factor R_L_ was calculated to evaluate adsorption favorability. For the initial concentration range of 70–170 mg·L^−1^, the R_L_ values ranged from 0.089 to 0.236 across all tested temperatures (298–318 K), all falling between 0 and 1, confirming thermodynamically favorable adsorption. The decreasing RL with increasing C_0_ indicates that higher initial NOR concentrations further enhance favorability.

The complete statistical indicators, including adj. R^2^, reduced χ^2^ (χ^2^__red_), SSE, and RMSE, are provided in Table S2.

#### 3.3.2. Adsorption Thermodynamics

To further explore the adsorption mechanism of P-CB for NOR, the influence of temperature on the adsorption process was investigated. The calculated thermodynamic parameters are presented in Table 5.

The negative ΔH° confirms that the overall adsorption process is exothermic. This indicates that the macroscopic driving force is dominated by physisorption, rather than chemisorption. This is broadly consistent with the expected decrease in Langmuir constant K_L_ with increasing temperature for an exothermic process (Table 4), noting that only three temperature points were measured. The positive ΔS° (55.2 J·mol^−1^·K^−1^) indicates increased disorder at the solid–liquid interface due to displacement of water molecules during adsorption. The negative ΔG° values confirm that the adsorption is highly spontaneous and thermodynamically favorable at all tested temperatures, with spontaneity increasing slightly at higher temperatures.

The thermodynamic and kinetic analyses provide complementary insights. Although the overall enthalpy change points to physisorption as the dominant energy contributor, the pseudo-second-order kinetic model (R^2^ > 0.9982) indicates that the rate-controlling step still involves chemical interactions. This is because the mesopore-dominated architecture ensures rapid physical diffusion, while the subsequent binding at specific active sites requires molecular orientation and electron redistribution, which proceed more slowly and thus govern the overall adsorption rate. Therefore, the adsorption of NOR onto P-CB follows a synergistic cascade mechanism: mesopore-accelerated physical transport as the prerequisite, P-anchored chemisorption as the kinetic lock, and π-π/electrostatic interactions as the supplementary stabilization. The thermodynamic and kinetic analyses are mutually complementary, together revealing a complete picture of the adsorption process.

### 3.4. Impact of Environmental Factors

#### 3.4.1. Influence of Initial Antibiotic Concentration

**Figure 8:**
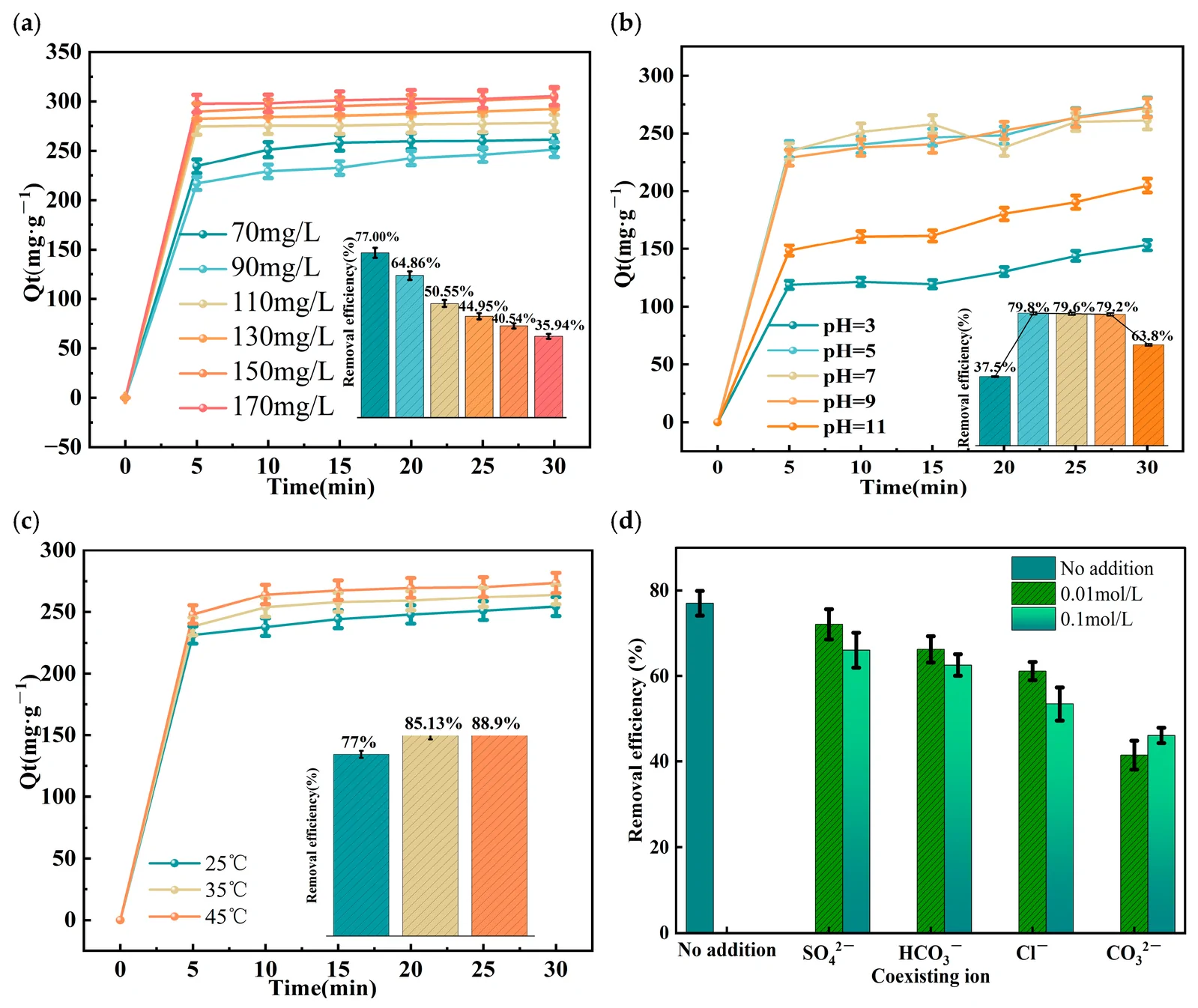
Factors affecting the adsorption of NOR onto P-CB: (**a**) pollutant concentration; (**b**) solution pH; (**c**) solution temperature; and (**d**) coexisting anions (adsorbent dosage = 10 mg, C_0_ = 70 mg·L^−^^1^).

The initial antibiotic concentration is a key parameter for evaluating the adsorption performance of modified biochar and its applicability in actual wastewater treatment. To investigate the influence of initial NOR concentration on the adsorption performance of P-CB, six concentrations ranging from 70 to 170 mg·L^−1^ were tested (Figure 8a). As the initial concentration increased, the equilibrium adsorption amount of NOR by P-CB increased from 251.35 mg·g^−1^ to 305.5 mg·g^−1^, while the removal rate at 30 min decreased from 77.00% to 35.94%. This phenomenon is attributed to the limited surface active sites of P-CB. At low concentrations, NOR molecules effectively occupy the available sites, resulting in a higher removal rate. At high concentrations, the available adsorption sites per unit mass of adsorbent become relatively insufficient, leaving excess NOR molecules unadsorbed. These findings are consistent with previous reports [5,56]. Notably, even at the highest initial concentration of 170 mg·L^−1^, the equilibrium adsorption amount of P-CB reached 305.5 mg·g^−1^, indicating that this material has good adsorption potential for treating wastewater with various NOR concentrations and can avoid resource waste from dilution.

#### 3.4.2. Effect of Solution pH

Solution pH is a key parameter that regulates the adsorption process by altering the morphology of the adsorbate and the surface properties of the adsorbent. As pH increased from 3 to 5, the equilibrium adsorption amount of NOR by P-CB rose sharply from 153.40 mg·g^−1^ to 272.85 mg·g^−1^. In the pH range of 5 to 9, the adsorption amount remained stably high. When pH rose to 12, the adsorption amount decreased to 204.90 mg·g^−1^ (Figure 8b).

The pH_PZC_ of P-CB was approximately 5.7 (Figure S1), below which the surface is positively charged and above which it is negatively charged.

This trend is closely related to the interplay between NOR morphology and the surface charge of the adsorbent. The pK_a1_ (carboxyl group) of NOR is approximately 6.3, and pK_a2_ (piperazine group) is approximately 8.4. Accordingly, NOR exists mainly as cations (NOR) at pH below 6.3, as zwitterions (NOR±) at pH 6.3–8.4, and as anions (NOR^−^) at pH above 8.4.

At pH < 5.7 (below pH_PZC_), the surface is positively charged, causing electrostatic repulsion with NOR^+^ cations and leading to low adsorption. As pH increases above 5.7, the surface becomes negatively charged. This generates electrostatic repulsion with NOR^−^ anions (pH > 8.4), and OH^_^ competes with NOR^_^ for adsorption sites, causing a decrease in adsorption capacity [57]. Specifically at pH 12, NOR is anionic, and strong electrostatic repulsion with the negatively charged P-CB surface, combined with OH^_^ competition, results in a significant drop in adsorption efficiency.

In conclusion, the adsorption of NOR by P-CB is dominated by electrostatic interactions, and the optimal adsorption performance occurs in the pH range of 5 to 9.

#### 3.4.3. Temperature Influencing Factors

To investigate the effect of reaction temperature on NOR adsorption by P-CB, experiments were conducted at 25 °C, 35 °C, and 45 °C with fixed adsorbent dosage and initial concentration (Figure 8c). The equilibrium adsorption amount and the amount adsorbed at each time point increased with temperature. After 30 min, the equilibrium adsorption amounts were 251.35 mg·g^−1^ (25 °C), 263.96 mg·g^−1^ (35 °C), and 273.66 mg·g^−1^ (45 °C). The temperature effect was already evident at early stages: at 5 min, the adsorption amounts were 231.37, 238.32, and 248.02 mg·g^−1^, respectively; at 10 min, they were 237.74, 253.84, and 264.21 mg·g^−1^, respectively.

This positive temperature dependence is attributed to two factors. First, higher temperature accelerates the thermal motion of NOR molecules, increasing the collision frequency with active sites on the P-CB surface and thus promoting adsorption [5]. Second, the increase in adsorption amount with temperature may be attributed to enhanced molecular thermal motion and reduced solution viscosity at higher temperatures, which facilitate mass transfer and increase the collision frequency between NOR molecules and the active sites on P-CB [5]. This temperature-dependent adsorption behavior does not necessarily imply an endothermic overall process; rather, it reflects the kinetic enhancement of diffusion and surface interactions at elevated temperatures.

#### 3.4.4. The Influence of Ionic Strength

To investigate the effects of coexisting salts on NOR adsorption by P-CB, four inorganic salts (Na_2_SO_4_, NaHCO_3_, NaCl, and Na_2_CO_3_) were tested at two concentrations (0.01 and 0.1 mol·L*^−^*^1^) with deionized water as a blank. As shown in Figure 8d, all salts inhibited NOR adsorption, but the concentration dependence varied by ion.

At 0.01 mol·L*^−^*^1^, the NOR removal rates followed Na_2_SO_4_ (72.07%) > NaCl (66.24%) > NaHCO_3_ (61.13%) > Na_2_CO_3_ (41.51%). Thus, the inhibition strength at low concentration was CO_3_^2−^ > HCO_3_^−^ > Cl^−^ > SO_4_^2−^. At 0.1 mol·L^−1^, the order changed: NaHCO_3_ (67.54%) > Na_2_SO_4_ (66.02%) > NaCl (53.47%) > Na_2_CO_3_ (46.14%), giving an inhibition order of CO_3_^2−^ > Cl^−^ > SO_4_^2−^ > HCO_3_^−^.

The mechanisms differ among anions. CO_3_^2−^ exhibited the strongest inhibition at both concentrations. This is attributed to two factors: (i) hydrolysis of CO_3_^2−^ raises the solution pH, shifting NOR speciation toward anions (consistent with the pH effect in Section 3.4.2, where adsorption decreases above pH 9); (ii) divalent CO_3_^2−^ competes more strongly for adsorption sites than monovalent anions [1,58,59]. Cl^−^ showed strong concentration dependence: the removal rate dropped sharply from 66.24% to 53.47% as concentration increased, the largest decline among all ions, indicating significant competitive adsorption at high Cl^−^ concentration. SO_4_^2−^ caused relatively mild interference; at 0.01 mol·L*^−^*^1^ its removal rate (72.07%) was close to the blank, and only moderate inhibition occurred at 0.1 mol·L*^−^*^1^. HCO_3_^−^ had the weakest inhibition at 0.1 mol·L*^−^*^1^ (highest removal rate, 67.54%), but at 0.01 mol·L*^−^*^1^ its inhibition was stronger than that of SO_4_^2−^ and Cl^−^. This behavior may be related to its weaker competitive ability at higher concentration, possibly due to changes in solution pH or speciation.

#### 3.4.5. Regeneration and Reusability

The reusability of P-CB was evaluated over three consecutive cycles using 0.1 mol·L^−1^ HCl as the desorption agent (Figure S2). The adsorption capacity decreased from 250.38 mg·g^−1^ (fresh) to 234.40, 221.88, and 186.49 mg·g^−1^ after the first, second, and third cycles, corresponding to retention rates of 93.6%, 88.6%, and 74.5%, respectively. The gradual decline is attributed to the irreversible occupation of strong chemisorption sites by NOR molecules and minor mass loss during washing and drying. Despite this decrease, P-CB retained over 74% of its initial capacity after three cycles, demonstrating satisfactory reusability for potential practical applications.

### 3.5. Summary of Adsorption Mechanism

The adsorption of NOR onto P-CB proceeds via a sequential cascade: mesopore-accelerated diffusion serves as the physical prerequisite; P-anchored chemisorption acts as the primary driving force; and π-π and electrostatic interactions provide auxiliary stabilization.

**Figure 9:**
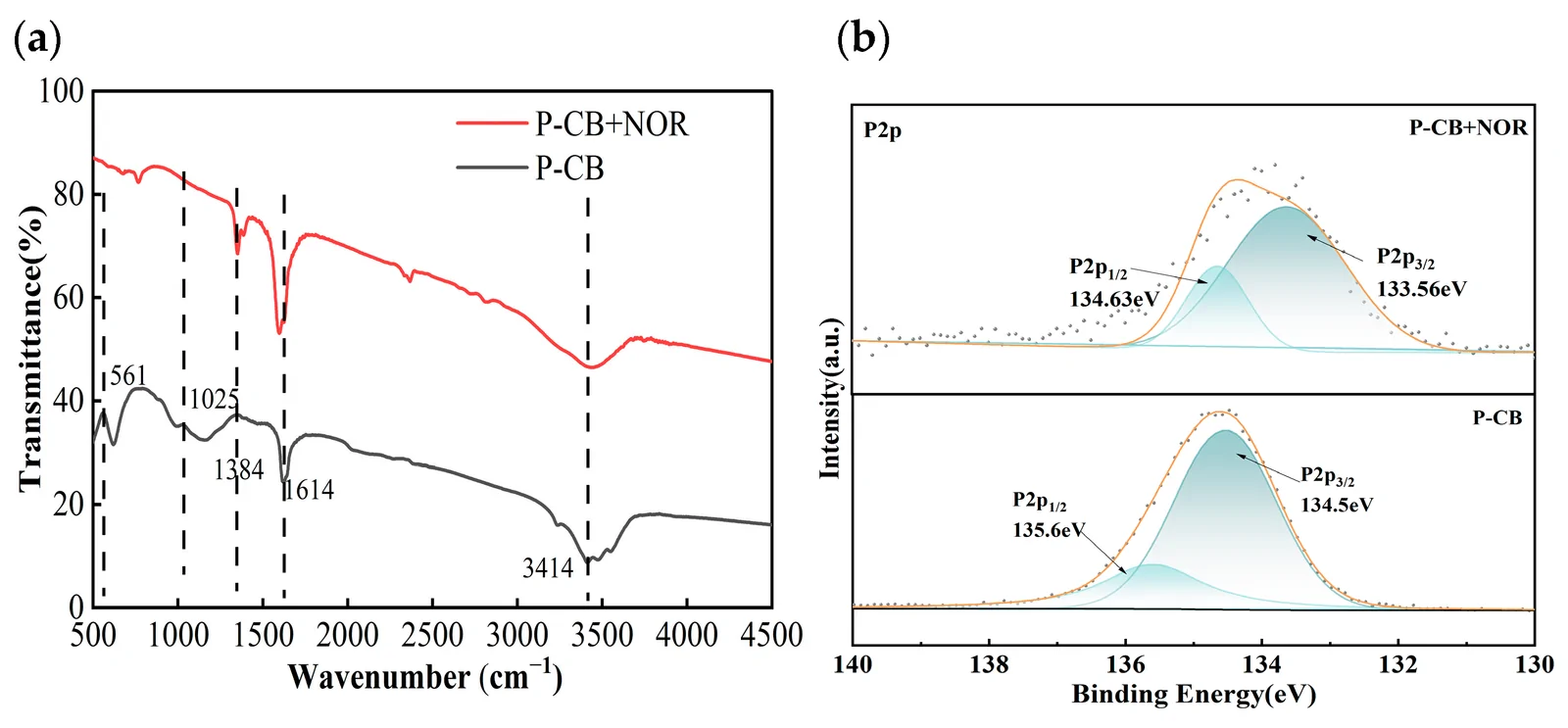
(**a**) FTIR spectra and (**b**) XPS P2p spectra of P-CB before and after NOR adsorption.

Post-adsorption spectroscopic evidence: To directly verify the chemical interactions, post-adsorption FTIR and XPS analyses were performed. The FTIR spectrum (Figure 9a) reveals weak NOR characteristic peaks in the 561–1025 cm^−1^ region [60], while the O–H stretching band at 3414 cm^−1^ decreases significantly after adsorption, indicating hydrogen bond formation between surface functional groups and NOR. The post-adsorption XPS analysis (Figure 9b) provides complementary evidence: In the figure, the scattered points represent the raw experimental data, the red solid line is the overall fitted envelope, and the deconvoluted peaks are shown in light blue (assigned to the P2p_1/2_ component) and dark blue (assigned to the P2p_3/2_ component). The P2p_1/2_ and P2p_3/2_ peaks shift negatively by ~0.95 eV, indicating increased electron density around phosphorus atoms and confirming that P-containing groups bind with NOR through hydrogen bonding [60,61].

Role of mesopores and phosphorus: Although P-doping drastically reduces the BET specific surface area, the average pore diameter expands to 14.25 nm, minimizing intraparticle diffusion resistance and enabling rapid initial uptake (>80% within 5 min). Quantitatively, the surface P content follows the order P-CB (11.10%) > P-WB (8.67%) > P-BB (7.24%), which correlates positively with the mesopore contribution (P-CB: 37.6%; P-WB: 24.6%; P-BB: 15.7%) and the adsorption capacity at 30 min (P-CB: 217.84; P-WB: 194.46; P-BB: 184.63 mg·g^−1^). These correlations confirm that phosphorus plays a dual role: etching the carbon framework to create mesopores and anchoring functional groups as chemisorption sites.

π-π, electrostatic, and isotherm implications: The graphitic domains, evidenced by the enhanced (002) peak in XRD and C=C bonds in XPS, act as π-electron donors for π-π interactions with the quinolone ring of NOR, showing good resistance to ionic interference (Section 3.4.4). The pH-dependent adsorption is rationalized by pH_PZC_ (5.7). At pH 5–9, favorable electrostatic conditions maximize adsorption, while outside this range, electrostatic repulsion reduces capacity. The temperature-dependent isotherm fitting further supports the transition from adsorbate–adsorbent interactions to homogeneous monolayer chemisorption at higher temperatures.

**Figure 10:**
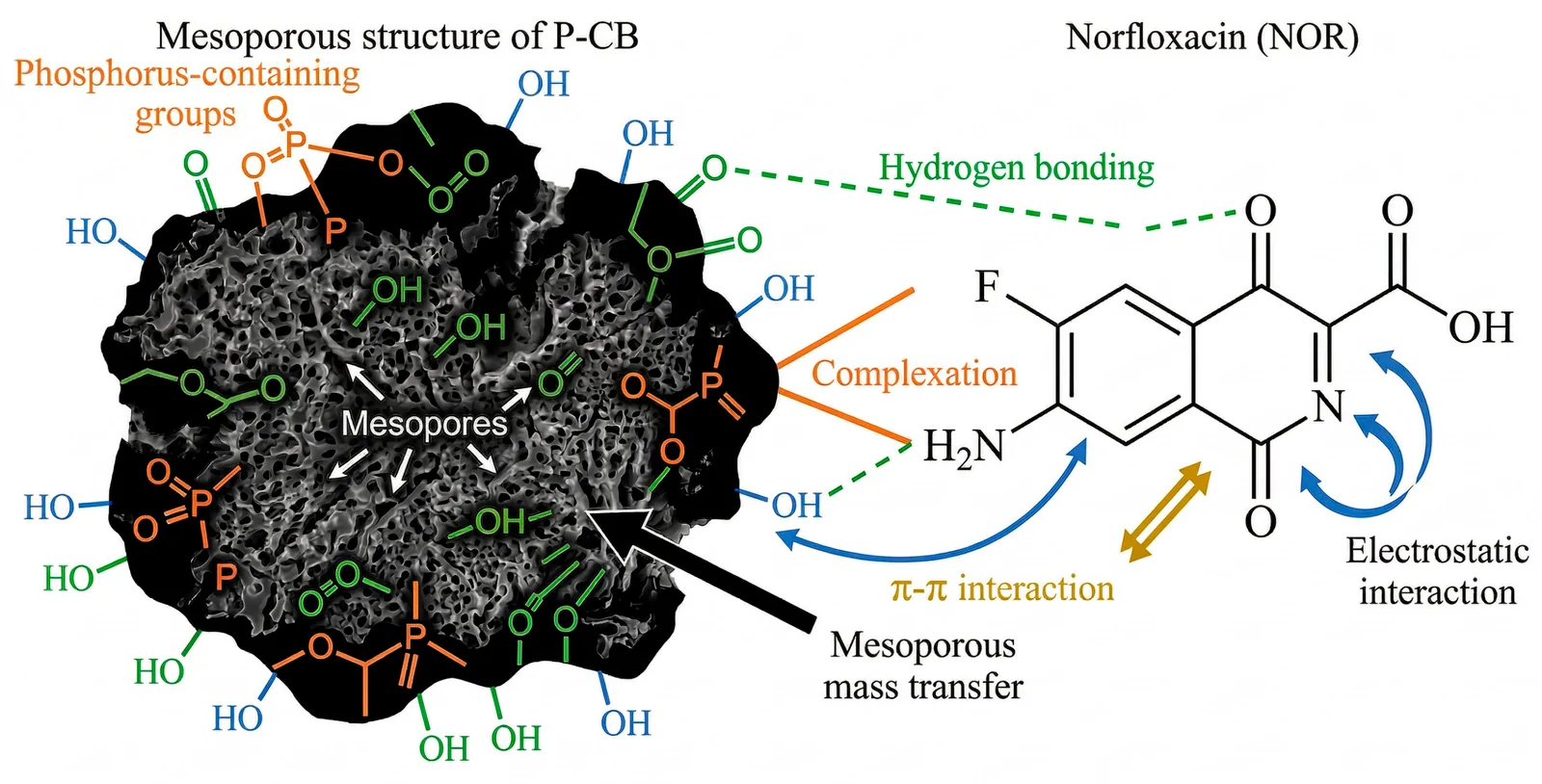
Adsorption mechanism of NOR removal by P-CB.

Synergistic cascade: The synergy arises from a three-step cascade. Mesopores rapidly deliver NOR molecules; P-anchored sites provide strong chemical capture; and π-π/electrostatic interactions offer supplementary stabilization. Without mesopores, the chemical sites would be buried in inaccessible micropores; without these sites, mesopores would only provide weak physical retention. This mesopore-enabled accessibility compensates for the loss of total surface area, offering a new design paradigm for biochar adsorbents targeting macromolecular pollutants. The mechanism adsorption diagram for the summary is shown in Figure 10.

## 4. Conclusions

(1) High-temperature (800 °C) phosphoric acid activation fundamentally restructured the pore architecture of biochar without causing dramatic additional mass loss compared to unmodified biochar. Although the BET specific surface area decreased dramatically (by 90–95% for P-CB and P-BB), the original microporous skeleton was transformed into a mesopore-dominated structure (average pore diameter 12–15 nm). This counterintuitive outcome, sacrificing surface area to create efficient mass-transfer channels, significantly enhanced the accessibility of norfloxacin molecules to active sites. Among the three P-doped biochars, corn straw-based P-CB exhibited the best adsorption performance, achieving 87.14% removal within 30 min (C_0_ = 50 mg·L^−1^) and a maximum adsorption capacity of 305.5 mg·g^−1^, outperforming P-WB and P-BB.
(2) Adsorption kinetics followed the pseudo-second-order model (R^2^ > 0.9982), confirming that chemisorption dominates the rate-controlling step. Isotherm data fitted the Freundlich model (1/n = 0.232–0.322), indicating heterogeneous surface sites with strong affinity. Thermodynamic parameters (ΔH° = –7.86 kJ·mol^−1^, ΔG° < 0) verified an exothermic, spontaneous process, indicating that the macroscopic driving force is dominated by physisorption, while the pseudo-second-order kinetics confirm that the rate-controlling step still involves chemical interactions at the P-anchored active sites. The synergistic mechanism combines mesopore-accelerated physical diffusion, π-π interactions from the graphitized carbon skeleton, and P-anchored chemical interactions.
(3) Solution pH significantly affected adsorption: the optimal range was 5–9 (adsorption capacity 251–273 mg·g^−1^), while strongly acidic (pH 3) or alkaline (pH 12) conditions sharply reduced performance, confirming the role of electrostatic interactions. Coexisting anions exerted mild inhibitory effects, with CO_3_^2−^ showing the strongest competition and SO_4_^2−^ the weakest.
(4) This study demonstrates that high-temperature P-doping, which prioritizes mesopore architecture, graphitic π-electron density, and phosphorus-containing functional groups over raw specific surface area, provides a new design paradigm for biochar adsorbents targeting macromolecular pollutants. Importantly, the comparable solid yield between P-CB and unmodified CB at 800 °C confirms that phosphorus primarily acts as a structural and chemical modifier rather than a mass-preserving agent. Real wastewater validation and systematic phosphorus leaching experiments are recommended for future studies to further assess the practical applicability and environmental safety of P-doped biochar.

## Figures and Tables

**Table 1:** Pore structure parameters of pristine and phosphorus-doped biochar samples.

Biochar	BET Specific Surface Area (m^2^·g^−1^)	Micropore Area (m^2^·g^−1^)	Micropore Volume (cm^3^·g^−1^)	Average Pore Diameter (nm)	Total Pore Volume (cm^3^·g^−1^)
CB	393.08	348.58	0.15	3.31	0.21
WB	345.43	285.01	0.12	3.74	0.20
BB	748.18	667.74	0.27	1.94	0.36
P-CB	23.56	14.70	0.001	14.25	0.11
P-WB	54.16	13.53	0.06	12.97	0.20
P-BB	61.31	9.63	0.00	11.94	0.22

**Table 2:** Adsorption kinetic model parameters.

Pollutant Concentration mg·L^−1^	*q_e,_*_exp_	Pseudo-First-Order Kinetic Model			Pseudo-Second-Order Kinetic Model			
	mg·g^−1^	K_1_	R^2^	*q*_e,cal_ (mg·g^−1^)	h (mg·g^−1^·min^−1^)	K_2_	R^2^	*q_e,cal_* (mg·g^−1^)
70	251.35	0.0927	0.9527	241.42	242.3	0.0037	0.9982	255.87
90	261.35	0.1567	0.9316	258.82	265.1	0.0038	0.9999	264.12
110	278.05	0.0687	0.8876	275.88	279.3	0.0036	0.9996	278.56
130	292.15	0.0618	0.8999	287.65	291.8	0.0034	0.9997	292.97
150	305.1	0.0642	0.9326	298.54	304.1	0.0033	0.9993	303.56
170	304.5	0.0719	0.8719	301.76	306.7	0.0033	0.9998	304.87

**Table 3:** Parameters of the intraparticle diffusion model.

Initial Concentration (mg·L^−1^)	K_ip,1_	C_1_	R_1_^2^	K_ip,2_	C_2_	R_2_^2^	K_ip,3_	C_3_	R_3_^2^
70	18.246	193.549	0.9982	6.582	231.088	0.9255	1.572	252.532	0.8495
90	13.117	187.617	0.9993	10.267	196.353	0.9937	3.71	225.811	0.9297
110	2.917	272.447	0.9854	0.991	271.934	0.9525	0.934	270.649	0.9056
130	2.383	276.335	0.9859	1.998	278.293	0.9594	0.053	284.171	0.9368
150	3.833	280.779	0.9993	3.621	281.411	0.9982	2.441	286.767	0.9944
170	3.466	285.317	0.9826	0.977	287.255	0.9687	0.216	297.963	0.7242

**Table 4:** Fitting parameters of adsorption isotherm models.

Temperature/K	Langmuir				Freundlich			Temkin		
	q_m_	K_L_ (L·mg^−1^)	R^2^	R_L_	K_F_ (mg^1−1/n^·L^1/n^·g^−1^)	1/n	R^2^	bT (J·mol^−1^)	AT (L·mg^−1^)	R^2^
298	360.4275	0.0601	0.9764	0.192	107.503	0.314	0.9855	39.07	1.3839	0.9932
308	502.1406	0.0463	0.9422	0.236	97.631	0.322	0.9764	24.54	0.5621	0.9768
318	517.64	0.0494	0.9766	0.224	85.3895	0.232	0.9786	24.39	0.5866	0.9885

**Table 5:** Thermodynamic parameters of NOR adsorption onto P-CB.

Temperature/K	$K^{{}^{\circ}}$/(Dimensionless)	$\Delta G^{{}^{\circ}}$/(KJ·mol^−1^)	ΔH°/(KJ·mol^−1^)	ΔS°/(J·mol^−1^·K^−1^)
298	1.92 × 10^4^	−24.4		
308	1.48 × 10^4^	−24.6	−7.86	55.2
318	1.58 × 10^4^	−25.6		

## Data Availability

The original contributions presented in the study are included in the article/Supplementary Materials. Further inquiries can be directed to the corresponding author.

## Supplementary Materials

The following supporting information can be downloaded at: https://www.mdpi.com/article/10.3390/ma19173584/s1, Table S1: Statistical indicators for kinetic model fitting (pseudo-first-order and pseudo-second-order) at different initial NOR concentrations. Table S2: Statistical indicators for isotherm model fitting (Langmuir, Freundlich, and Temkin) at different temperatures. Figure S1: Point of zero charge (pH_PZC_) determination of P-CB by the pH drift method. Figure S2: Regeneration performance of P-CB for NOR adsorption over three consecutive cycles. Reference [62] is cited in the Supplementary Materials.
