# Supplementary material for: Overriding Surface Area Limitation: Mesopore-Driven Norfloxacin Adsorption on High-Temperature P-Doped Biochar
Source: Materials (Basel). 2026 Aug 24;19(17):3584. doi: 10.3390/ma19173584 (PMC13566808; doi:10.3390/ma19173584)
Supplement: Supplementary file 1 [file materials-19-03584-s001.zip › materials-4378549-supplementary.pdf]

Supporting Information

Section S1. Adsorption Model Equations

Kinetic models

Pseudo-first-order (PFO) model:

$$q_t=q_e\left(1-e^{-tk_1}\right) \qquad (S1)$$

where q_t ($mg\cdot g^{-1}$) and q_e ($mg\cdot g^{-1}$) are the adsorption capacities at time t and at equilibrium, respectively; k_1 (min^{-1}) is the pseudo-first-order rate constant.

Pseudo-second-order (PSO) model:

$$q_t=\frac{q_e^2k_2t}{q_ek_2t+1} \qquad (S2)$$

where k_2 ($g\cdot mg^{-1}\cdot min^{-1}$) is the pseudo-second-order rate constant.

Elovich model:

$$q_t=\frac{1}{\beta}\ln(1+\alpha\beta t) \qquad (S3)$$

where α ($mg\cdot g^{-1}\cdot min^{-1}$) is the initial adsorption rate constant, and β ($g\cdot mg^{-1}$) is the desorption constant.

Intraparticle diffusion model:

$$q_t=K_{id}t^{\frac{1}{2}}+C \qquad (S4)$$

where K_{id} ($mg\cdot g^{-1}\cdot min^{-1/2}$) is the intraparticle diffusion rate constant, and C is the boundary layer thickness constant.

Isotherm models

Langmuir model:

$$q_e=\frac{q_mK_LC_e}{1+K_LC_e} \qquad (S5)$$

where q_m ($mg\cdot g^{-1}$) is the maximum monolayer adsorption capacity, and K_L ($L\cdot mg^{-1}$) is the Langmuir adsorption constant.

Freundlich model:

$$q_e=K_FC_e^{\frac{1}{n}} \qquad (S6)$$

where K_F ($mg^{1-1/n}\cdot L^{1/n}\cdot g^{-1}$) is the Freundlich adsorption constant, and 1/n is the adsorption intensity factor.

Sips model:

$$q_e=\frac{Q_mK_sC_e^{n_s}}{1+K_sC_e^{n_s}} \qquad (S7)$$

where K_s is the Sips equilibrium constant, and n_s is the Sips exponent.

Temkin model:

$$q_e=\frac{RT}{b_t}\ln(A_TC_e) \qquad (S8)$$

where R (8.314 J·mol^{-1}·K^{-1}) is the universal gas constant, T (K) is the absolute temperature, b_T (J·mol^{-1}) is the Temkin constant related to adsorption heat, and A_T (L·g^{-1}) is the Temkin equilibrium binding constant.

Thermodynamic equations

It should be emphasized that the apparent distribution coefficient Kd = q_e/C_e (with units of L·g^{-1}) is dimensional and thus cannot be directly used for thermodynamic calculations [1]. Instead, the dimensionless thermodynamic equilibrium constant K° was derived from the Langmuir isotherm constant K_L (L·mg^{-1}) following the conversion procedure recommended by Tran et al. [1]:

$$K° = K_L \times 1000 \times M_W \qquad (S9)$$

where Mw = 319.34 g·mol^{-1} is the molar mass of norfloxacin. This conversion is valid when q_e and C_e are expressed in mg·g-1 and mg·L^{-1}, respectively, and the standard conce ntration C° is taken as 1 mol·L^{-1} (with activity coefficient $\gamma \approx 1$).

The standard Gibbs free energy change was then calculated as:

$$\Delta G° = -RT\ln K° \qquad (S10)$$

The standard enthalpy change (ΔH°) and entropy change (ΔS°) were obtained from the linear van't Hoff equation:

$$\ln K° = -\frac{\Delta H°}{R} \times \frac{1}{T} + \frac{\Delta S°}{R} \qquad (S11)$$

where R is the universal gas constant (8.314 J·mol^{-1}·K^{-1}), and T is the absolute temperature (K).

Table S1. Statistical indicators for kinetic model fitting (pseudo-first-order and pseudo-second-order) at different initial NOR concentrations.

Model	C_0 (mg·L^{-1})	adj R^2	Reduced χ^2	SSE	RMSE (mg·g^{-1})
PFO	70	0.921	7.136	28.54	2.671
	90	0.886	57.438	229.75	7.580
	110	0.813	1.195	4.78	1.093
	130	0.833	8.403	33.61	2.899
	150	0.888	17.765	71.06	4.215
	170	0.787	4.543	18.17	2.131
PSO	70	0.997	1.164	4.66	1.079
	90	0.999	17.762	71.05	4.214
	110	0.999	0.633	2.53	0.796
	130	0.999	5.037	20.15	2.244
	150	0.999	7.769	31.08	2.787
	170	0.999	1.957	7.83	1.399

Table S2. Statistical indicators for isotherm model fitting (Langmuir, Freundlich, and Temkin) at different temperatures.

Model	C_0 (mg·L^{-1})	adj R^2	Reduced χ^2	SSE	RMSE (mg·g^{-1})
Freundlich	298	0.978	16.939	67.76	4.116
	308	0.965	83.761	335.04	9.152
	318	0.968	85.390	341.56	9.241
Langmuir	298	0.965	12.687	50.75	3.562
	308	0.913	34.189	136.75	5.847

	318	0.965	36.644	146.55	6.053
Temkin	298	0.990	7.687	30.75	2.773
	308	0.965	119.961	479.85	10.954
	318	0.983	43.967	175.87	6.631

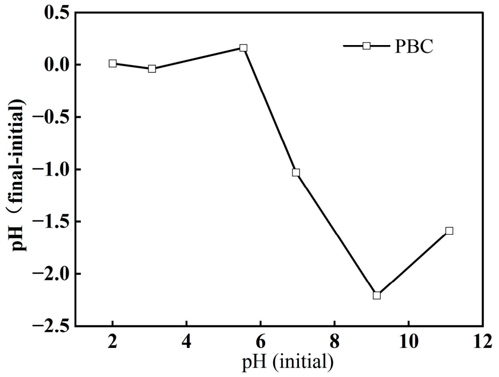

Figure S1. Point of zero charge (pH_{PZC}) determination of P-CB by the pH drift method

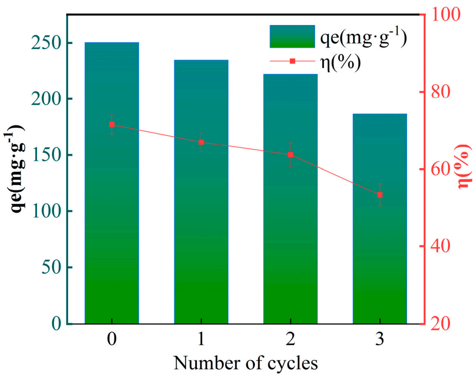

Figure S2. Regeneration performance of P-CB for NOR adsorption over three consecutive cycles.

[1]Tran, H.N.; Lima, E.C.; Juang, R.S.; Bollinger, J.C.; Chao, H.P. Thermodynamic parameters of liquid-phase adsorption process calculated from different equilibrium constants related to adsorption isotherms: A comparison study. J. Environ. Chem. Eng. 2021, 9, 106674.
